# Cortactin as a Target for FAK in the Regulation of Focal Adhesion Dynamics

**DOI:** 10.1371/journal.pone.0044041

**Published:** 2012-08-29

**Authors:** Alok Tomar, Christine Lawson, Majid Ghassemian, David D. Schlaepfer

**Affiliations:** 1 Moores University of California San Diego Cancer Center, University of California San Diego, La Jolla, California, United States of America; 2 Department of Chemistry and Biochemistry, University of California San Diego, La Jolla, California, United States of America; University of Birmingham, United Kingdom

## Abstract

**Background:**

Efficient cell movement requires the dynamic regulation of focal adhesion (FA) formation and turnover. FAs are integrin-associated sites of cell attachment and establish linkages to the cellular actin cytoskeleton. Cells without focal adhesion kinase (FAK), an integrin-activated tyrosine kinase, exhibit defects in FA turnover and cell motility. Cortactin is an actin binding adaptor protein that can influence FA dynamics. FAK and cortactin interact, but the cellular role of this complex remains unclear.

**Principal Findings:**

Using FAK-null fibroblasts stably reconstituted with green fluorescent protein (GFP) tagged FAK constructs, we find that FAK activity and FAK C-terminal proline-rich region 2 (PRR2) and PRR3 are required for FA turnover and cell motility. Cortactin binds directly to FAK PRR2 and PRR3 sites via its SH3 domain and cortactin expression is important in promoting FA turnover and GFP-FAK release from FAs. FAK-cortactin binding is negatively-regulated by FAK activity and associated with cortactin tyrosine phosphorylation. FAK directly phosphorylates cortactin at Y421 and Y466 and over-expression of cortactin Y421, Y466, and Y482 mutated to phenylalanine (3YF) prevented FAK-enhanced FA turnover and cell motility. However, phospho-mimetic cortactin mutated to glutamic acid (3YE) did not affect FA dynamics and did not rescue FA turnover defects in cells with inhibited FAK activity or with PRR2-mutated FAK that does not bind cortactin.

**Conclusions:**

Our results support a model whereby FAK-mediated FA remodeling may occur through the formation of a FAK-cortactin signaling complex. This involves a cycle of cortactin binding to FAK, cortactin tyrosine phosphorylation, and subsequent cortactin-FAK dissociation accompanied by FA turnover and cell movement.

## Introduction

Cell migration plays important roles during development and contributes to pathological processes such as tumor invasion and metastasis [1]. Cell movement is initiated by events including the formation of leading-edge membrane protrusions and integrin-associated focal adhesions (FAs) [2]. FAs link the intracellular filamentous-actin (f-actin) cytoskeleton to the extracellular matrix and serve as points of traction for tension generation [3]. Leading edge cell projections are stabilized by FA formation and the severing of f-actin linkages can also trigger FA turnover [4]. Various intracellular proteins act to regulate FA assembly and disassembly as this is an important control point for cell movement. One of these proteins is actin binding adaptor protein cortactin [5], [6].

Cortactin is a modular protein with a N-terminal acidic domain that binds to Arp2/3 involved in actin nucleation, followed by multiple tandem cortactin repeats that bind f-actin, a proline-rich region containing tyrosine phosphorylation sites, and a C-terminal Src-homology 3 (SH3) domain connecting cortactin to other actin-associated proteins such as N-WASP [7]. Cortactin contributes to FA turnover upon growth factor stimulation of MEFs [8] and cortactin serine/threonine as well as tyrosine phosphorylation are linked to changes in actin dynamics [9]. Cortactin tyrosine phosphorylation occurs at Y421, Y466, and Y482, is mediated by multiple tyrosine kinases [10], and results in SH2-mediated adaptor protein binding to phosphorylated cortactin [11]. In vitro, cortactin tyrosine phosphorylation alters f-actin cross-linking activity. In cells, cortactin tyrosine phosphorylation is associated with enhanced cell migration and invadopodia formation [5], [12]. Tyrosine to phenylalanine substitutions in cortactin inhibit FA turnover whereas tyrosine to glutamic acid substitutions may increase FA turnover dynamics [9]. How signaling complexes with cortactin are temporally assembled to mediate changes in actin polymerization affecting FA turnover remains unresolved.

Focal adhesion kinase (FAK) is a cytoplasmic tyrosine kinase activated by integrin and growth factor receptors in the control of FA dynamics and cell movement [13]. FAK is comprised of an N-terminal FERM domain, a central catalytic domain, three proline-rich regions (PRR) that are sites of SH3 domain binding [14], and a C-terminal FA-targeting domain connecting FAK to integrins [15]. FAK knockout or knockdown results in cells with motility defects and slow FA turnover kinetics [16]. Pharmacological or genetic inhibition of FAK results in FA turnover defects [17], [18]. FAK phosphorylates various FA and actin regulatory proteins controlling FA dynamics during cell motility [15], [19]. These targets include Src [20], p190RhoGAP [21], p130Cas [22], paxillin [23], N-WASP [24], and α-actinin [25]. Additionally, FAK autophosphorylation at Y397 creates a SH2 binding site for Src-family tyrosine kinases and the generation of a FAK-Src signaling complex [20], [26].

In addition to the importance of intrinsic FAK activity in promoting cell motility and increasing FA turnover, point mutations within FAK C-terminal PRR domains prevent FAK function in promoting motility [27], [28] and cell invasion [29]. Interestingly, FAK PRR mutations do not prevent integrin-stimulated FAK activation and phosphorylation at Y397 [28]. Thus, it has been hypothesized that the FAK-Src complex may phosphorylate proteins bound to FAK PRR regions leading to signal generation controlling cell motility. One of these targets is the adaptor protein p130Cas involved in promoting FA disassembly potentially via effects on matrix degradation [30]–[32]. Other FAK PRR binding proteins such as GRAF (GTPase regulator associated with FAK) and PSGAP (PH- and SH3-domain containing RhoGAP protein) [33], [34] have been proposed to link FAK to actin cytoskeletal alterations. Cortactin is also a FAK PRR binding protein participating in signaling events associated with bacterial uptake [35], [36]. Recent studies have linked the FAK-cortactin complex to increased tumor cell radiotherapy resistance [37] and in the control of cell motility [38], [39].

Although FAK is implicated in promoting Src-enhanced cortactin tyrosine phosphorylation at FAs [38], it is unclear whether this requires a direct binding interaction with FAK or intrinsic FAK activity. Moreover, it remains undetermined whether FA remodeling and motility defects of FAK-null cells involve alterations in cortactin tyrosine phosphorylation. Here, we evaluate FAK-null fibroblasts reconstituted with wildtype (WT), kinase-inactive (K454R), or FAK PRR2/PRR3-mutated green fluorescent protein (GFP) tagged FAK. We find that direct cortactin SH3 domain-mediated binding to FAK PRR2/PRR3 domains and FAK phosphorylation of cortactin promote increased FA turnover and cell motility. Analyses of cortactin phosphorylation site mutants revealed that tyrosine phosphorylation was essential for FAK-enhanced turnover of FAs, but that cortactin tyrosine to glutamic acid substitutions were insufficient to promote FA turnover in FAK-KD and FAK-PRR2 mutated fibroblasts. Together, these studies support the importance of a FAK-cortactin signaling complex in the regulation of FA dynamics and cell motility.

## Results

### FAK PRR2- and PRR3-inactivating Mutations Prevent Cell Motility and FA Turnover

**Figure 1:**
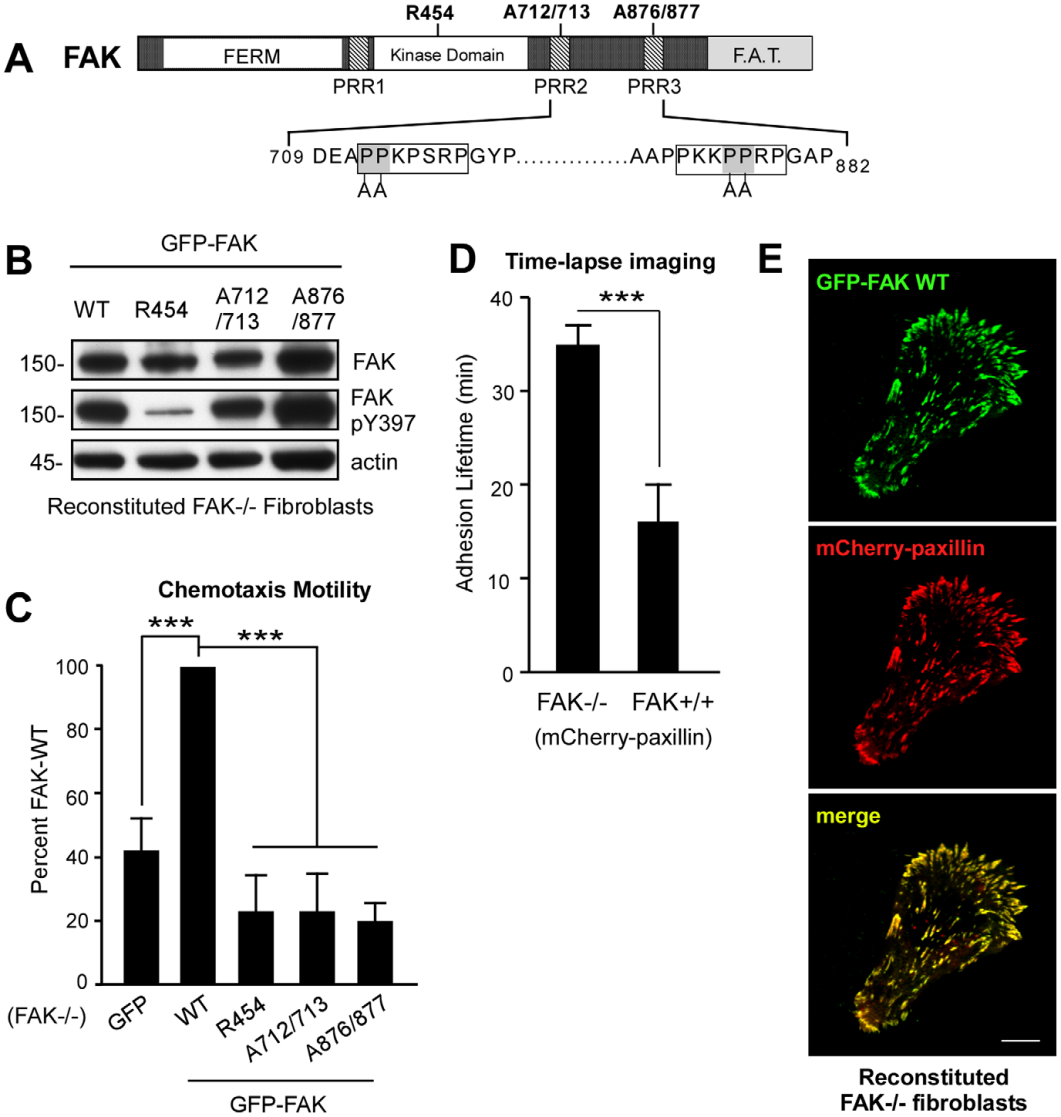
FAK activity and C-terminal PRR regions are required for cell motility. (A) Schematic of FAK comprised of an N-terminal FERM domain, a central catalytic domain, three proline-rich regions (PRR) that are sites of SH3 domain binding, and a C-terminal FA-targeting domain (F.A.T) connecting FAK to integrins. Point-mutations are indicated that disrupt catalytic activity (lysine to arginine, R454), and SH3 domain binding to PRR2 (proline to alanine, A712/713) or PRR3 (proline to alanine, A876/877). (B) Lysates from the indicated GFP-FAK reconstituted FAK-/− MEFs showing GFP-FAK expression, FAK Y397 phosphorylation (pY397), and actin by immunoblotting. (C) Chemotaxis cell motility on FN-coated Millicell chambers over 4 h. Cell number is expressed as a percent of GFP-FAK WT (normalized to 100, ± SEM, ***p<0.001) from 3 independent experiments. (D) FA lifetime determination by counting sequential frames of mCherry paxillin fluorescence above background using time-lapse confocal microscopy. Images were acquired at 2 min intervals over 60 min in FAK−/− and FAK+/+ MEFs stimulated with growth media containing 50 ng/ml EGF. Data is the mean lifetime ± SEM of 125–150 FAs from at least 5 different cells (***p<0.001). (E) Co-localization of GFP-FAK and mCherry-paxillin at FAs in MEFs plating on FN-coated (10 µg/ml) coverslips for 60 min. Scale is 10 µm.

Over-expression, knockdown, and the creation of FAK knockout mouse embryo fibroblasts (MEFs) have established the importance of FAK as a multi-functional signaling protein promoting cell movement [15], [19]. Kinase-inactive (R454) knockin or exon deletion studies support the conclusion that FAK activity and FAK Y397 phosphorylation enhance cell motility [18], [40]. Transient FAK re-expression studies in FAK−/− MEFs have also identified the importance of FAK PRR regions in promoting cell motility [28]. As FAK−/− MEFs stably reconstituted with GFP-FAK exhibit motility properties identical to normal MEFs [41], comparisons were made with FAK−/− MEFs stably-reconstituted with GFP-FAK WT or GFP-FAK constructs containing mutations disrupting kinase activity (R454), PRR2 (proline to alanine, A712/713), or PRR3 (proline to alanine, A876/877) (Fig. 1A and B). Alanine substitutions in PRR2 (DEAPPKPSRPGYP to DEAAAKPSRPGYP) or in PRR3 (AAPPKKPPRPGAP to AAPPKKAARPGAP) disrupt SH3 binding interactions [14], [42], but do not block FAK Y397 phosphorylation in cells (Fig. 1B).

In evaluating the motility properties of GFP-only or GFP-FAK WT reconstituted FAK−/− MEFs, cell movement in Boyden chamber chemotaxis assays was significantly-increased by FAK-WT (Fig. 1C, p<0.001). FAK WT chemotaxis was significantly greater than FAK−/− MEFs equally- expressing FAK R454, PRR2 mutated FAK A712/713, or PRR3 mutated FAK A876/877 (Fig. 1C, p<0.001). It is known that FAK−/− MEF movement is hampered by slow FA turnover [16]. Paxillin localizes to FAs and can be used as marker of FA dynamics. Transfection of FAK−/− and FAK+/+ MEFs with fluorescent mCherry-paxillin and time-lapse imaging of cells revealed significantly longer adhesion lifetime within FAK−/− compared to FAK+/+ MEFs (Fig. 1D, p<0.001). Co-transfection of mCherry-paxillin into GFP-FAK WT MEFs revealed extensive co-localization at FAs (Fig. 1E). As previous quantitative studies have documented that FA assembly-disassembly rates for GFP-paxillin and GFP-FAK are the same [43], real-time imaging analyses of GFP-FAK can be used as a marker of FA dynamics.

**Figure 2:**
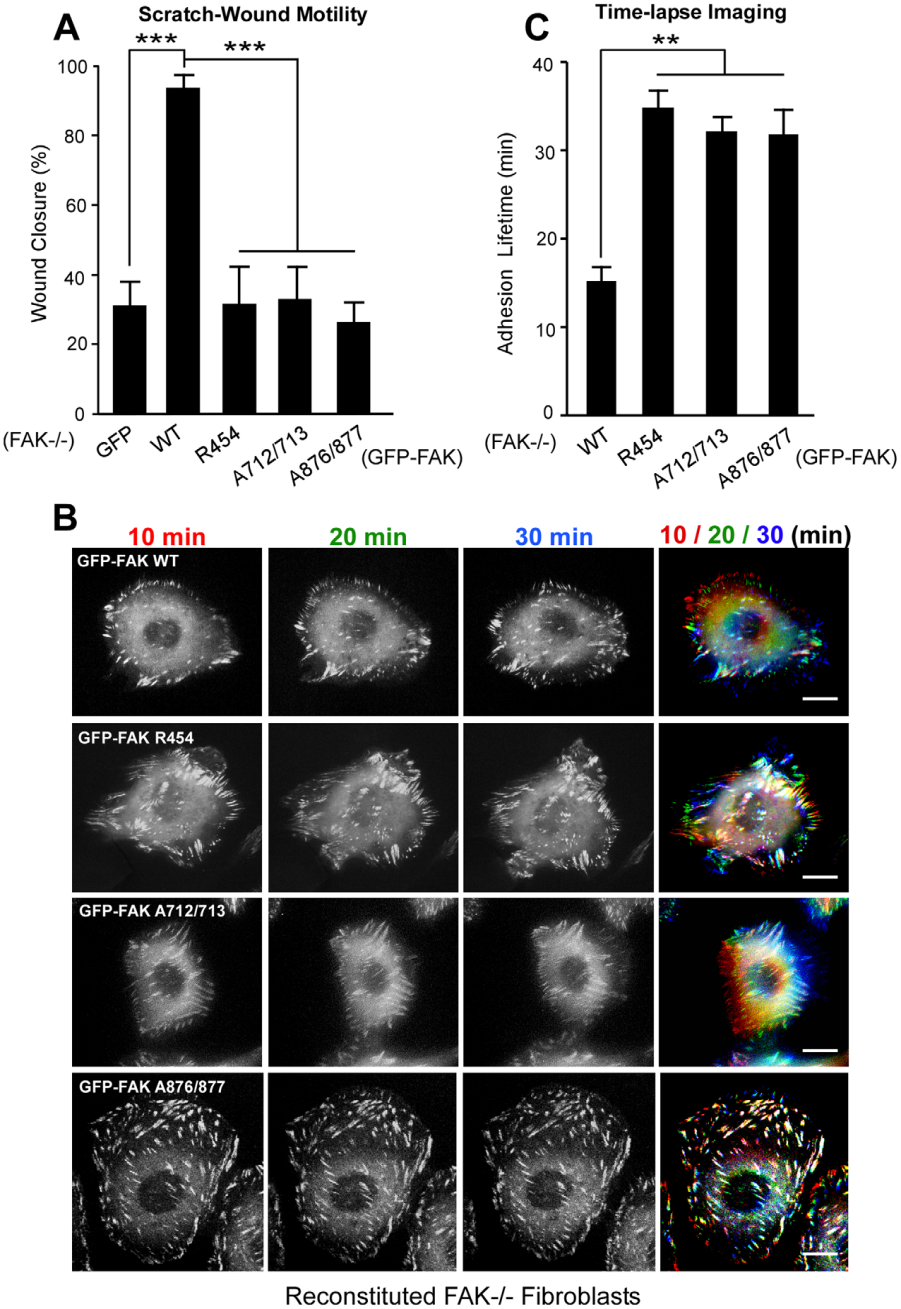
FAK activity and C-terminal PRR regions are required for FA turnover. (A) Scratch wound assay performed with FAK−/− fibroblasts expressing GFP or the indicated GFP-FAK constructs. Closure percentage was calculated by the change in area between 2 and 10 h. Experimental values are presented as ± SEM from 3 independent experiments (***p<0.001). (B) Representative image montage (10 to 30 min) from live-cell spinning disc confocal microscopy of FA-localized GFP-FAK after growth media supplemented with 50 ng/ml EGF stimulation. A merged image from the 10/20/30 min time points was pseudo-colored red (10 min), green (20 min) and blue (30 min) respectively to illustrate GFP-FAK localization over time. White regions indicate GFP-FAK localization overlap at 10 to 30 min. Scale is 10 µm. (C) Adhesion lifetime was determined by counting the number of sequential frames (2 min intervals within a 60 min time-lapse) of GFP-FAK FA-associated fluorescence above background. Data is the mean lifetime ± SEM of 150–200 FAs from at least 5 different cells from each of the indicated GFP-FAK reconstituted FAK−/− MEFs (**p<0.01, compared to FAK-WT).

Time-lapse imaging revealed that GFP-FAK WT reconstituted MEFs closed scratch wounds significantly faster than GFP-only or GFP-FAK R454, FAK A712/713, or FAK A876/877 reconstituted FAK−/− MEFs (Fig. 2A, p<0.001). In random motility assays, time-lapse analysis of single cells and superimposition of images at 10 min (red), 20 min (green), and 30 min (blue) to create a montage over a 30 min period revealed rapid GFP-FAK WT movement within cells corresponding to active FA dynamics (Fig. 2B and Video S1). In contrast, GFP-FAK R454, FAK A712/713, or FAK A876/877 reconstituted MEFs exhibited an extended residency time of the GFP-FAK mutants at both leading and trailing-edge FAs as depicted by white-colored FAs in superimposed images of cells (Fig. 2B and Video S2). Quantitation revealed up to 2-fold increased lifetime of GFP-FAK residency at FAs for FAK R454, FAK A712/713, and FAK 876/877 mutants compared to FAK WT (Fig. 2C, p<0.01). Together, these results support the notion that mutations disrupting either FAK activity or SH3 target protein binding interactions with FAK C-terminal PRR regions prevent cell motility associated with decreased cellular FA dynamics.

### FAK PRR2- or PRR3 Domain Mutations Disrupt Cortactin Binding

**Figure 3:**
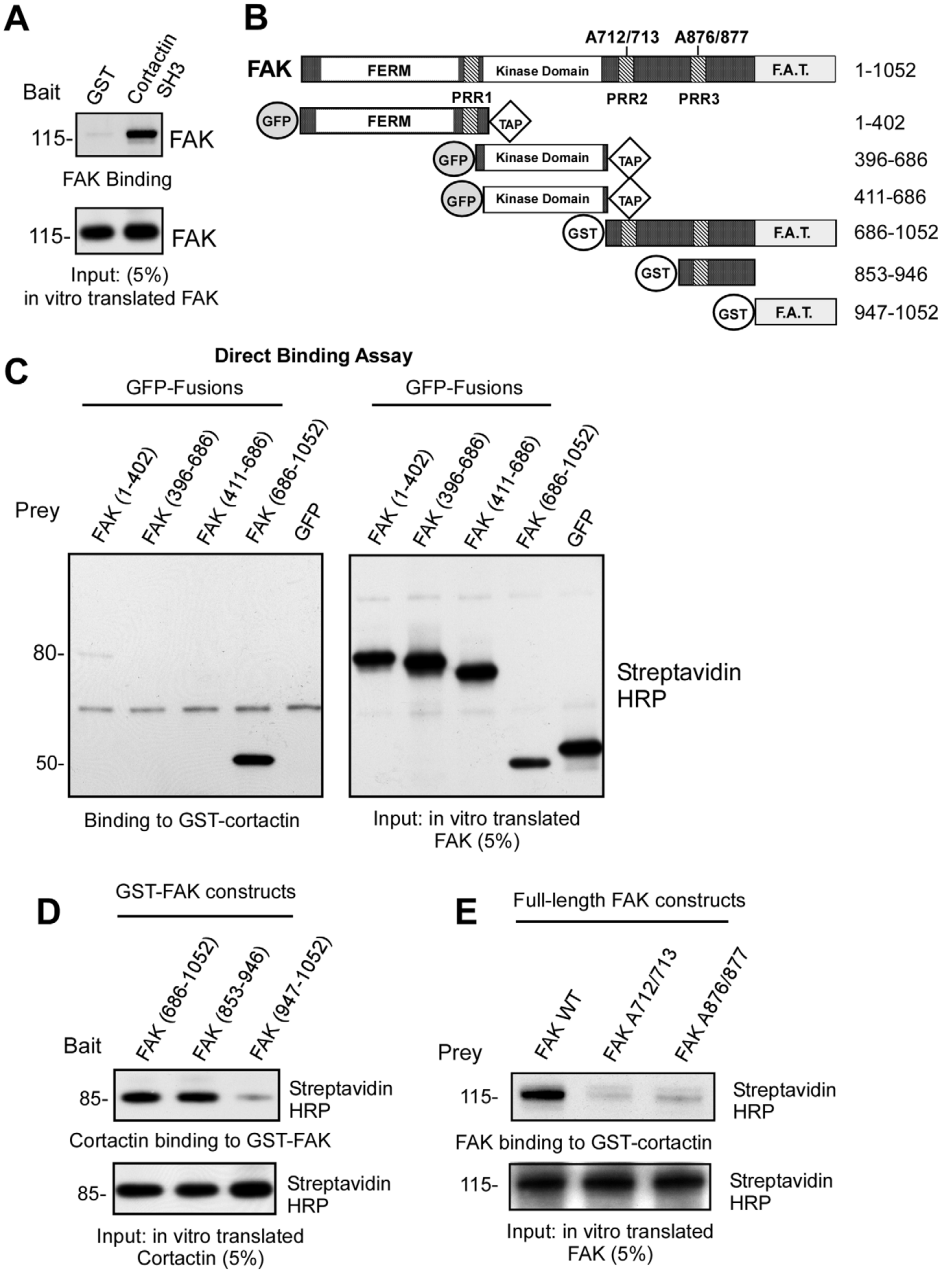
FAK PRR2 and PRR3 regions are direct binding sites for cortactin. (A) Cortactin SH3 domain binds FAK as determined by GST or GST-cortactin SH3 domain pulldown assays using in vitro translated FAK protein. FAK immunoblotting shows binding and 5% of input. (B) Schematic of FAK containing an N-terminal FERM domain, three PRR (PRR1-PRR3) sites, a central kinase domain, and a C-terminal FA targeting region. Point-mutations are indicated that disrupt PRR2 (A712/713) and PRR3 (A876/877). The indicated FAK regions (below) were used as bait or prey in direct binding assays as either GFP, C-terminal TAP, or GST fusion proteins. (C) FAK C-terminal domain binds cortactin. GFP fusions of FAK 1-402, FAK 396–686, and FAK 411–686 with a C-terminal TAP tag or non-tagged FAK 686–1052 were in vitro translated in the presence of biotin-lysine and used in a direct binding assay with GST or GST-cortactin attached to beads. Streptavidin-HRP analyses show the amount of FAK bound or 5% of prey material used in the binding assay. (D) FAK PRR3 region binds cortactin. In vitro translated full-length cortactin was incubated with GST-FAK (853–946) or GST-FAK (947–1052) in a direct binding assay. Streptavidin-HRP analyses show the amount of cortactin bound or 5% of prey material used in the binding assay. (E) FAK PRR2 and PRR3 are individually required for cortactin binding. GFP-fusions of FAK WT, A713/713, or A876/877 were in vitro translated and incubated with GST-cortactin in a direct binding assay. Streptavidin-HRP analyses show the amount of GFP-FAK bound or 5% of prey material used in the binding assay.

Recent studies have shown that FAK and cortactin interact in cells and that this binding is dependent upon the integrity of the cortactin SH3 domain and FAK C-terminal PRR regions [36]–[38]. To determine whether this represents a direct binding interaction, full-length FAK was in vitro translated and incubated with purified glutathione-S-transferase (GST) or GST-cortactin SH3 domain fusion protein (Fig. 3A). Strong binding between the cortactin SH3 domain and FAK was observed and these assays were extended using various fragments of FAK encompassing the N-terminal, kinase, or C-terminal domains and full-length recombinant GST-cortactin (Figs. 3B and C). Only the FAK C-terminal region (686–1052) encompassing PRR2 and PRR3 regions bound directly to cortactin (Fig. 3C).

Within the FAK C-terminal region, residues 853–946 encompassing PRR3 but not the FAK focal adhesion targeting domain (FAT, 947–1052) as GST fusion proteins were sufficient to mediate a direct binding interaction with in vitro translated cortactin (Fig. 3D). Lastly, full-length FAK WT, FAK A712/713, and FAK A876/877 were in vitro translated and used in a direct binding assay with GST-cortactin (Fig. 3E). Mutation of either PRR2 or PRR3 prevented cortactin binding. Together, these results support the direct binding of the cortactin SH3 domain to both FAK PRR2 and PRR3 regions. As previously observed for other SH3-mediated binding partners of FAK such as p130Cas [44], mutation in one of two FAK C-terminal PRR motifs is sufficient to disrupt binding interactions under the stringent buffer conditions as used in these assays.

### Cortactin Expression is Important in Promoting FA Turnover

**Figure 4:**
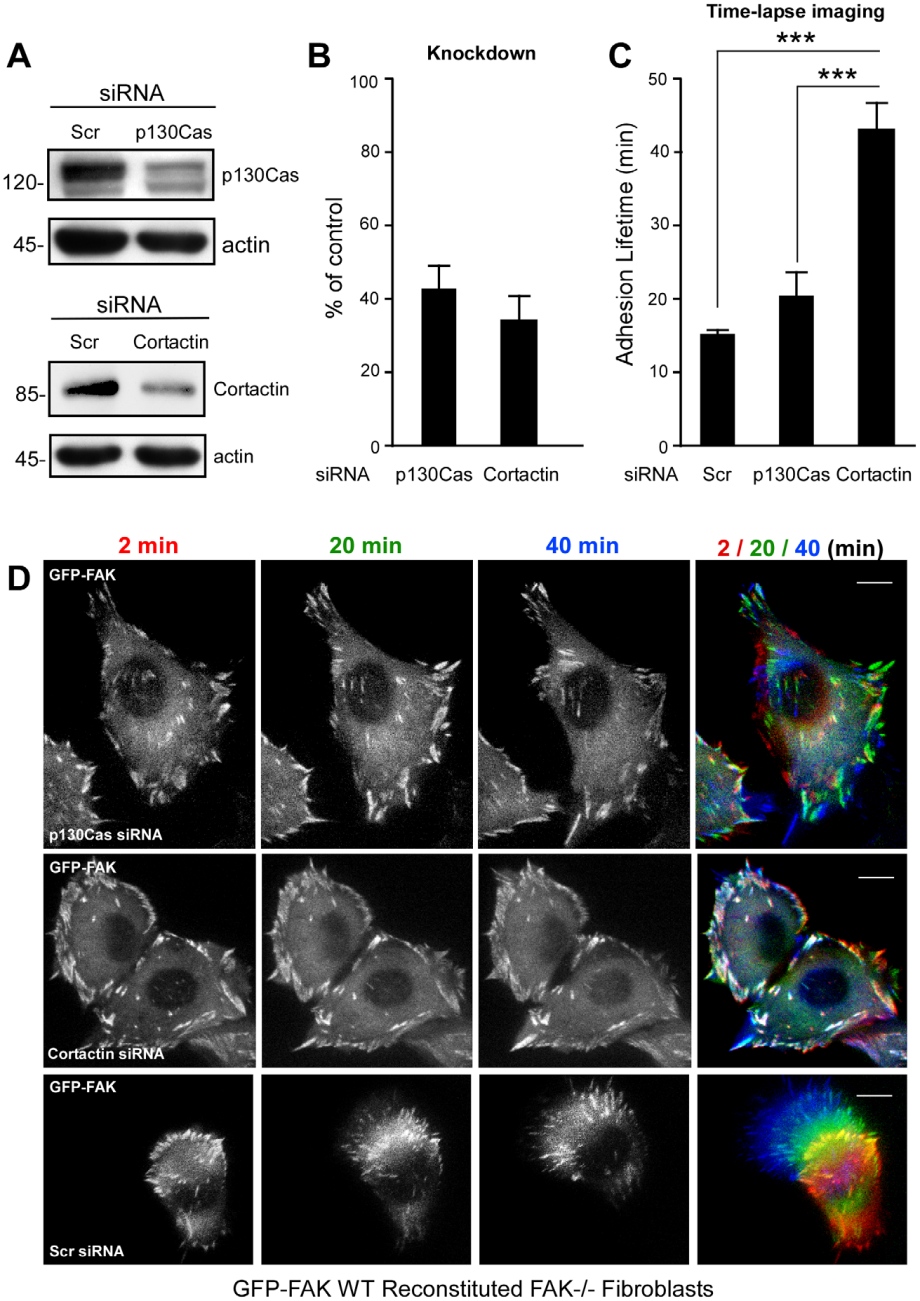
Cortactin knockdown results in decreased FA turnover. (A) GFP-FAK WT MEFs were transiently transfected with scrambled (Scr), cortactin, or p130Cas siRNA and protein levels determined after 48 h by immunoblotting. (B) Densitometry analyses of p130Cas and cortactin levels as a percent of Scr control. Values are means ±SD from two experiments. (C) Adhesion lifetime was determined by counting the number of sequential frames (2 min intervals from a 60 min time-lapse) for FA-associated GFP-FAK fluorescence above background. Data represent mean lifetime±SEM of 125–150 FAs from at least 5 different cells for Scr-, cortactin, or p130Cas siRNA-transfected GFP-FAK MEFs (***p<0.001). (D) Representative image montage (2 to 40 min) from live-cell spinning disc confocal microscopy of FA-localized GFP-FAK after growth media supplemented with 50 ng/ml EGF stimulation. As indicated, cells were transfected with control, anti-p130Cas, or anti-cortactin siRNA along with fluorescent marker (siGLO, not shown). A merged image from the 2/20/40 min time points was pseudo-colored red (2 min), green (20 min) and blue (40 min) respectively to illustrate GFP-FAK localization over time. White regions indicate GFP-FAK localization overlap at 2 and 40 min. Scale is 10 µm.

Recent studies using either cortactin-null or p130Cas-null MEFs have revealed a common phenotype of slower FA turnover after growth factor stimulation or at the leading edge of wounded cells, respectively [8], [31]. Since the SH3 domains of cortactin and p130Cas can bind FAK PRR2 and PRR3 domains, transient knockdown experiments were performed to determine the relative importance of cortactin or p130Cas in promoting FA turnover in GFP-FAK WT MEFs (Fig. 4). Transient transfection of cortactin- or p130Cas-specific siRNA resulted in 60 to 65 percent knockdown as determined by immunoblotting (Figs. 4A and B). For cell studies, siRNA-expressing cells were identified by co-transfection of a fluorescent cell marker (siGLO) and FA dynamics were evaluated by real-time imaging of GFP-FAK (Figs. 4C, 4D, and Video S3,S4,S5). Compared to scrambled siRNA (Scr)-expressing cells, p130Cas knockdown had only a small inhibitory effect on FA dynamics with an average lifetime extended from 15 to 22 min (Fig. 4C). Image superimposition revealed only a few white FAs as indicative of stable FAs within the 2–40 min imaging time period upon p130Cas siRNA transfection (Fig. 4D and Video S4). In contrast, cortactin knockdown dramatically inhibited GFP-FAK dynamics resulting in numerous white FAs in image superimposition analyses (Fig. 4D and Video S5). The mean GFP-FAK lifetime at FAs was extended from 15 to >40 min upon cortactin siRNA transfection and this was significantly different (p<0.001) from p130Cas knockdown (Fig. 4C). These results support the importance of cortactin in regulating GFP-FAK dynamics at FAs.

### FAK and Cortactin Binding is Negatively Regulated during MEF Adhesion to FN

**Figure 5:**
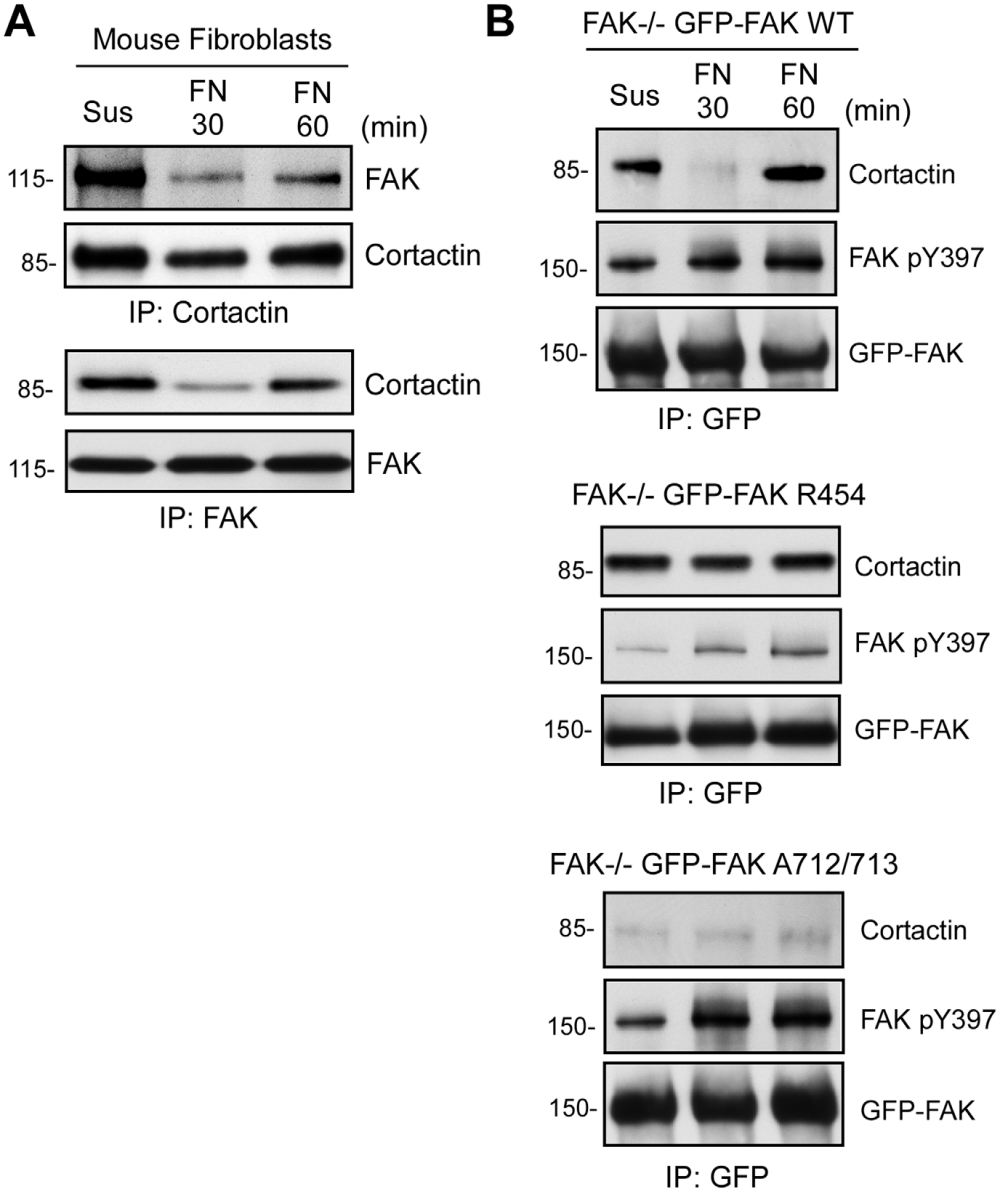
Regulation of FAK and cortactin binding by FN adhesion. (A) Lysates were made from MEFs held in suspension for 30 min or FN re-plated (30 and 60 min) and were analyzed by cortactin or FAK antibody immunoprecipitation (IP) followed by immunoblotting for cortactin and FAK. (B) Mutations disrupting FAK activity or within PRR2 either stabilize or prevent cortactin association with FAK by co-IP analyses, respectively. Lysates were made from the indicated GFP-FAK reconstituted MEFs held in suspension for 30 min or FN plated (30 and 60 min) and were analyzed by anti-GFP IPs followed by anti-cortactin, anti-FAK Y397 phosphorylation (pY397), and anti-GFP immunoblotting to determine the level of GFP-FAK.

Interactions between FAK and cortactin downstream of β1 integrin have been linked to cancer cell resistance to radiotherapy [37]. However, our analyses find that FAK and cortactin form a complex in MEFs held in suspension under conditions of limited β1 integrin signaling as determined by co-immunoprecipitation (co-IP) with antibodies to FAK, cortactin (Fig. 5A), or to GFP in FAK-reconstituted MEFs (Fig. 5B). Interestingly, the FAK-cortactin complex was only weakly detected in lysates of MEFs replated onto FN for 30 min (Fig. 5A and B). By 60 min on FN, FAK-cortactin binding increased (Fig. 5A and B). These results suggests that the formation of a FAK-cortactin complex is dynamic and may be negatively regulated by integrin signaling associated with MEF spreading on FN.

Moreover, FAK is maximally-activated at 30 min on FN [45] and genetic inhibition of FAK activity in FAK R454-reconstituted MEFs resulted in equal levels of FAK R454-cortactin complex formation in suspended and FN-replated cells (Fig. 5B). As expected, mutation of FAK PRR2 in GFP-FAK A712/713-reconstituted MEFs disrupted cortactin binding, but did not prevent FN-stimulated FAK tyrosine phosphorylation at Y397 (Fig. 5B).

**Figure 6:**
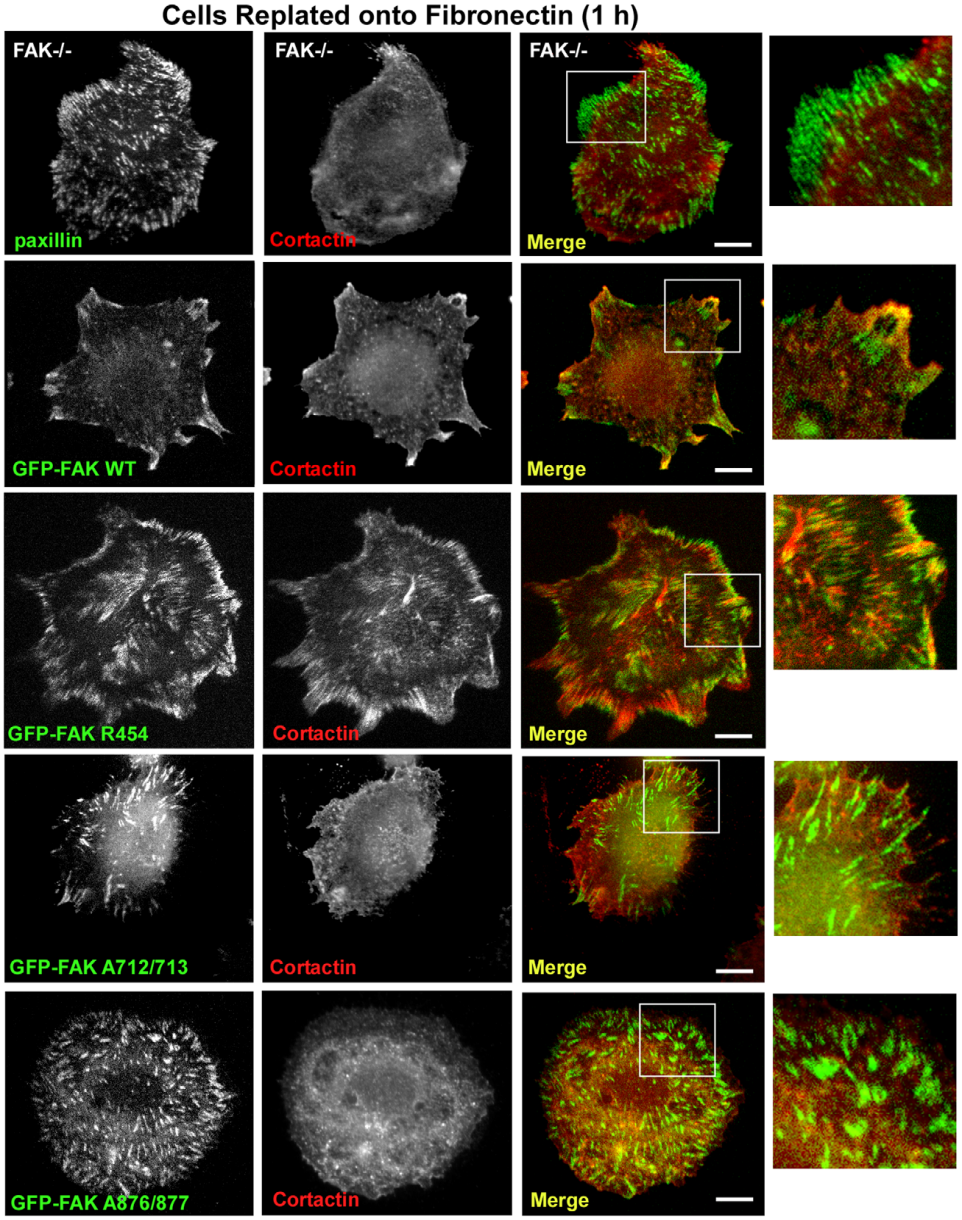
FAK PRR2 or FAK PRR3 regions are needed to promote transient cortactin co-localization with FAK at FAs. FAK−/− or the indicated GFP-FAK reconstituted MEFs were serum starved, held in suspension, FN replated for 60 min, and then analyzed for endogenous paxillin (green) or cortactin (red) immuno-staining. GFP-FAK was visualized by intrinsic fluorescence. Shown is GFP-FAK or paxillin and cortactin distribution within cells and merged images were used to evaluate co-localization (yellow). Scale is 10 µM. Inset, 4X enlargement of the corresponding box.

At 60 min on FN, paxillin is localized to FAs formed in FAK−/− MEFs whereas antibody staining for cortactin was not observed at these sites (Fig. 6). In FAK-reconstituted MEFs, partial co-localization of endogenous cortactin is detected with GFP-FAK WT and this co-localization at FAs is enhanced within GFP-FAK R454 MEFs upon adhesion to FN for 60 min (Fig. 6). Cortactin did not co-localize with GFP-FAK A712/713 or GFP-FAK A876/877 upon FN adhesion at 60 min, thus supporting the specificity of antibody staining. Together, these results confirm the importance of FAK C-terminal PRR domains in mediating cortactin binding and transient recruitment to FAs during the processes of cell spreading on FN. The association and co-localization of cortactin with FAK R454 supports the notion that intrinsic FAK activity negatively regulates cortactin binding to FAK through an undetermined mechanism. This regulatory connection is consistent with the formation of a cortactin-FAK complex in suspended cells where FAK activity is low.

### Cortactin is Phosphorylated by FAK

**Figure 7:**
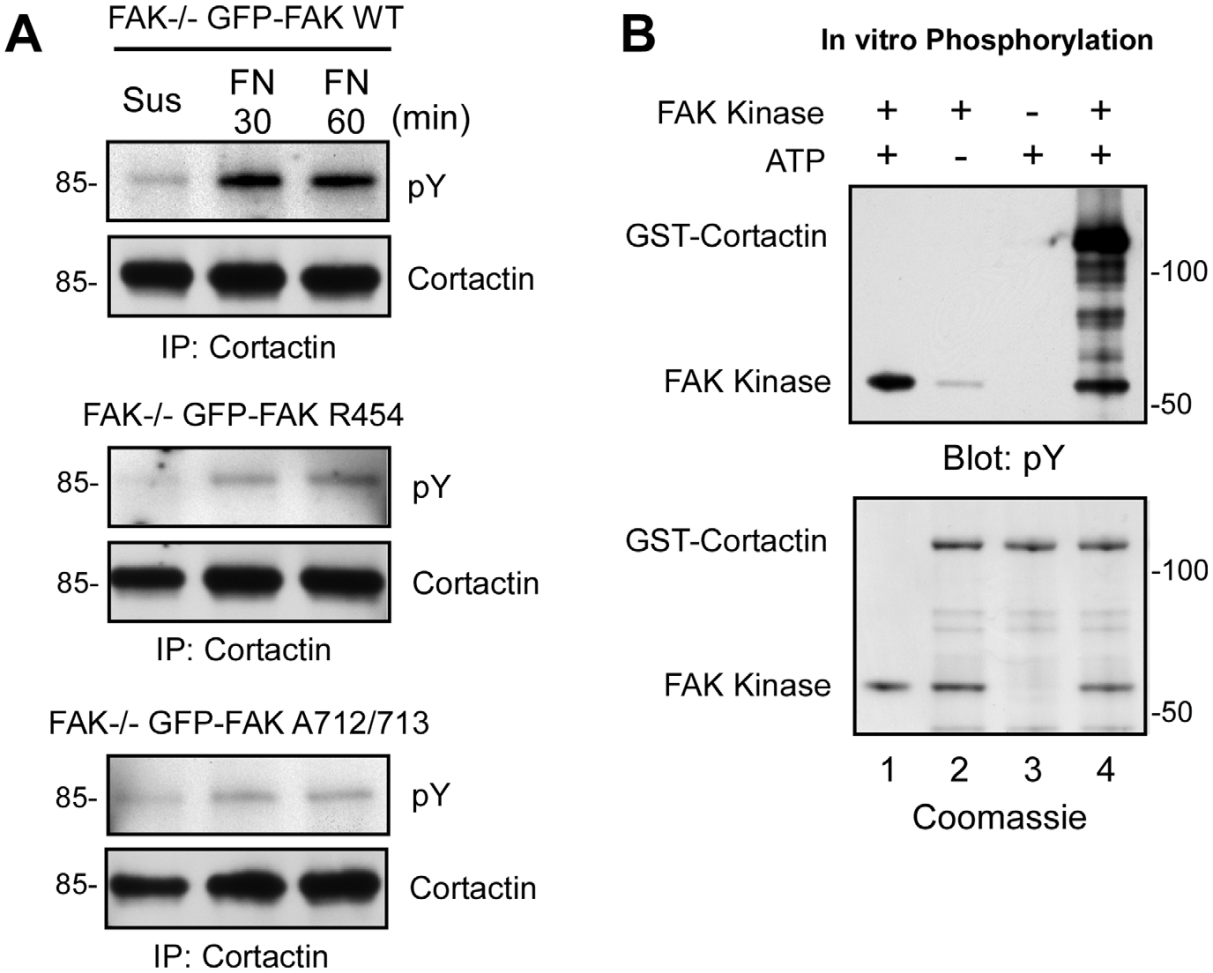
FAK tyrosine phosphorylation of cortactin during FN replating of MEFs. (A) Lysates were made from the indicated GFP-FAK reconstituted MEFs held in suspension for 30 min or FN plated (30 and 60 min) and were analyzed by anti-cortactin IPs followed by anti-phosphotyrosine (pY) and anti-cortactin immunoblotting. (B) In vitro kinase assays using recombinant GST-Cortactin incubated in the presence (lanes 1, 2 and 4) or absence (lane 3) of recombinant FAK kinase, or presence (lanes 1,3 and 4) and absence (lane 2) of ATP. Proteins were analyzed by anti-phosphotyrosine (pY) immunoblotting and by Coomassie staining.

Tyrosine phosphorylation of cortactin occurs upon cell adhesion to FN and this has been linked to Src and Abl/Arg tyrosine kinase activation [11]. To determine the role of FAK in this event, FAK WT, FAK R454, and FAK A712/713 reconstituted MEFs were either held in suspension or replated onto FN and cortactin tyrosine phosphorylation was analyzed by immunoblotting (Fig. 7A). As expected, no cortactin tyrosine phosphorylation was detected in suspended cells. In GFP-FAK WT MEFs, cortactin was phosphorylated within 30 min upon FN replating. However, cortactin tyrosine phosphorylation did not occur upon GFP-FAK R454 or GFP-FAK A712/713 MEF replating onto FN (Fig. 7A). These results support the notion that cortactin binding to FAK and intrinsic FAK activity are important in promoting cortactin tyrosine phosphorylation upon MEF adhesion to FN.

Cortactin can be phosphorylated by Src and Abl/Arg family protein-tyrosine kinases at Y421, Y466 and Y482 and this is correlated with alterations in lamellipodial or actin cytoskeletal dynamics [46], [47]. To determine if cortactin is a direct substrate of FAK, in vitro kinase assays were performed with purified GST-cortactin and recombinant FAK kinase domain (Fig. 7B). By using phospho-specific immunoblotting and mass spectrometry analysis of in vitro phosphorylated cortactin (Fig. S1), we found that FAK phosphorylated murine cortactin at analogous sites corresponding to Y421 and Y466. These results support the importance of FAK activity and direct cortactin binding to FAK for FN-stimulated cortactin tyrosine phosphorylation. Moreover, as recent studies have found that tyrosine to glutamic acid or aspartate mutations at cortactin Y421, Y466, and Y482 weaken binding interactions with FAK [36], [38], our results are consistent with the hypothesis that cortactin tyrosine phosphorylation may negatively regulate FAK binding during FN-stimulated cell spreading.

### Tyrosine to Phenylalanine Mutations within Cortactin Prevent FAK-mediated FA Turnover and Cell Motility

**Figure 8:**
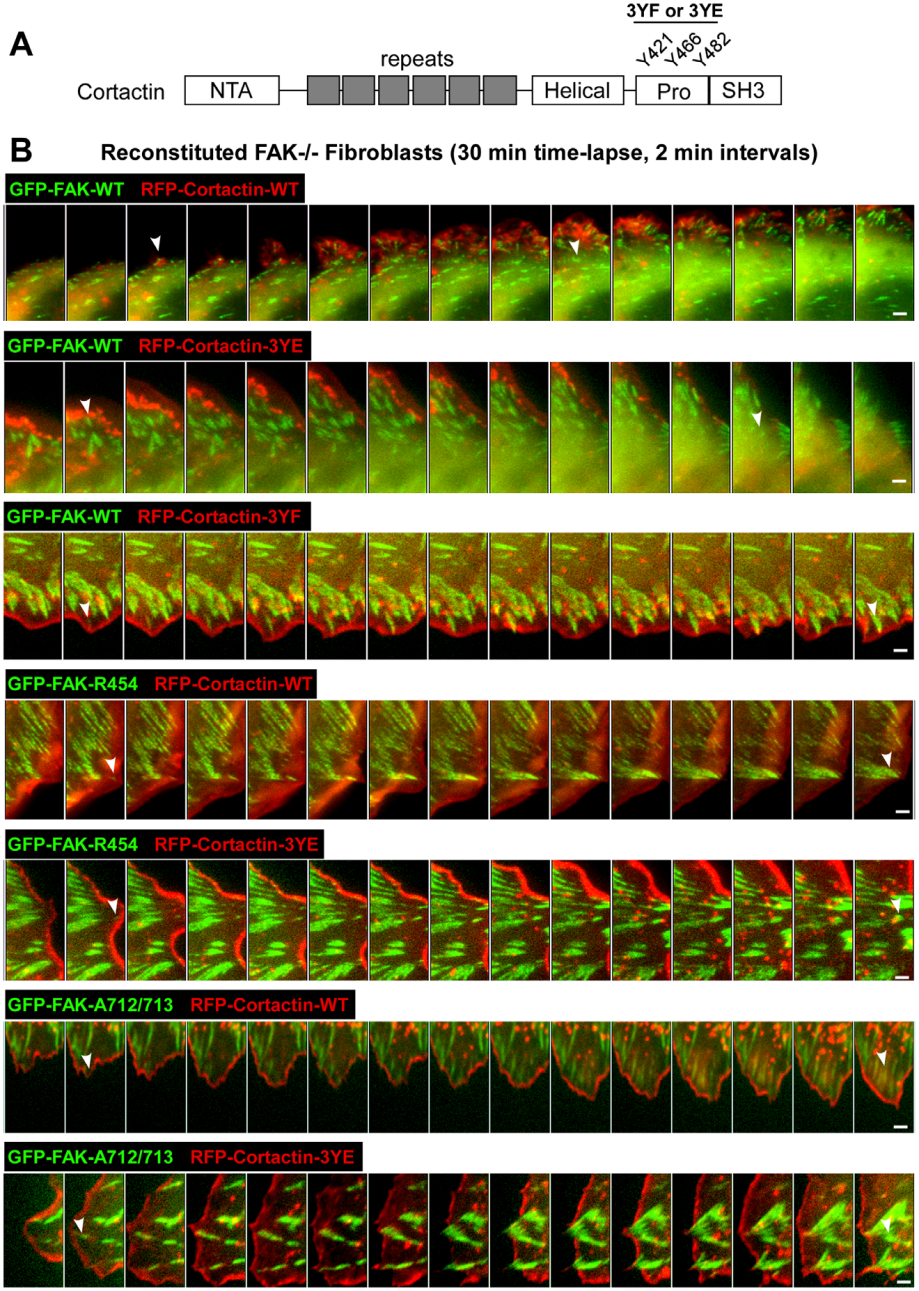
Cortactin tyrosine to phenylalanine (3YF) mutation prevents FAK-mediated cell migration and FA turnover. (A) Schematic of cortactin with an N-terminal acidic region (NTA), six cortactin tandem repeats, a helical region, a proline-rich region containing Y421, Y466, and Y482 phosphorylation sites (human numbering), and a carboxyl-terminal SH3 domain. (B) Reconstituted FAK−/− MEFs expressing GFP-FAK were transfected with RFP-cortactin WT, or RFP-cortactin with Y421, Y466, and Y482 mutated to phenylalanine (3YF) or mutated to glutamic acid (3YE). Representative images are shown as a kymograph of merged images extracted from live-cell spinning disc confocal microscopy after growth media supplemented with 50 ng/ml EGF stimulation (30 of 60 min time-lapse, 2 minute intervals). Images were pseudo-colored in red (RFP-cortactin) and in green (GFP-FAK) to illustrate GFP-FAK adhesion lifetime. White arrows indicate appearance of GFP-FAK in a given FA and when this particular FA disappears or remains within the cell region of interest. Scale is 1 µm.

Cortactin tyrosine phosphorylation is correlated with changes in cell motility, lamellipodia persistence, and FA dynamics [5]. Major sites of cortactin tyrosine phosphorylation are within a proline-rich region next to the C-terminal cortactin SH3 domain (Fig. 8A). Over-expression studies with cortactin Y421, Y466, and Y482 mutated to phenylalanine (3YF) or mutated to glutamic acid (3YE) can either prevent or enhance FA and cell edge protrusion dynamics, respectively [9], [11], [38].

**Figure 9:**
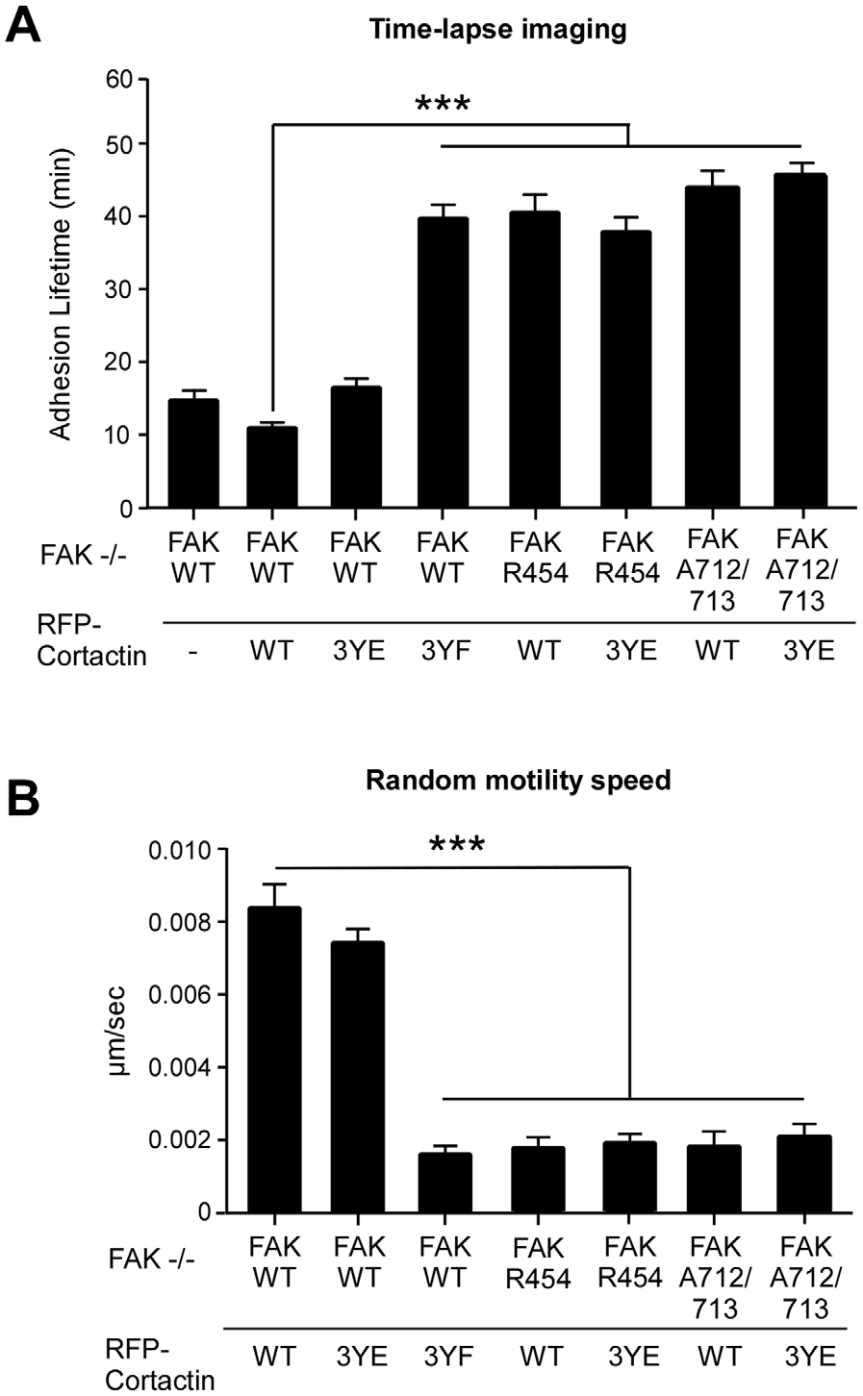
Cortactin tyrosine to phenylalanine (3YF) mutation inhibits FAK-mediated cell migration and FA turnover. FAK−/− MEFs reconstituted with GFP-FAK WT, GFP-FAK R454, or GFP-FAK A712/713 were co-transfected with RFP-cortactin WT, or RFP-cortactin Y421, Y466, and Y482 mutated to phenylalanine (3YF) or mutated to glutamic acid (3YE). (A) In MEFs over-expressing the indicated RFP-cortactin constructs, FA lifetime was determined by counting the number of sequential frames with GFP-FAK FA-associated fluorescence above background. Data from live-cell spinning disc confocal microscopy after growth media supplemented with 50 ng/ml EGF stimulation (2 min intervals from a 60 min time-lapse) represents the mean lifetime of at least 50 leading edge-associated FAs ± SEM from at least 12 different cells within the indicated GFP-FAK reconstituted FAK−/− MEFs (***p<0.001). (B) Cell migration speed in µm/sec was determined by cell tracking (n = 12 per group, values are means ±SD, ***p<0.001).

GFP-FAK re-expression in FAK−/− MEFs enhances FA turnover (Fig. 2) and cortactin knockdown slows FA turnover (Fig. 4). To evaluate the effects of cortactin tyrosine phosphorylation on FAK-mediated FA dynamics, red fluorescent protein (RFP) fusions of cortactin WT, 3YF, or 3YE were transfected into GFP-FAK WT reconstituted cells and confocal time-lapse imaging was performed (Fig. 8B). Compared to untransfected GFP-FAK WT MEFs with a mean FA lifetime of 15 min, RFP-cortactin WT expression resulted in slightly increased FA turnover kinetics with a mean lifetime of 11 min (Fig. 9A). Transient co-localization of GFP-FAK and RFP-cortactin was visualized within peripheral FAs (Fig. 8B and Video S6) and average cell speed was ∼0.008 µm/sec (Fig. 9B).

Co-localization of FAK WT and cortactin WT has been previously reported [38], however, co-localization of FAK with cortactin 3YE or 3YF remains uninvestigated. Transfection of RFP-cortactin 3YE resulted in slightly increased FA lifetime (17 min) compared to RFP-cortactin WT transfected cells (Fig. 9A), but this was equal to GFP-FAK WT MEFs not over-expressing cortactin. Notably, RFP-cortactin 3YE was strongly localized to cell protrusions and did not detectably co-localize with GFP-FAK WT at FAs (Fig. 8B and Video S7). RFP-cortactin 3YE expression was accompanied by slightly reduced cell speed compared to cells expressing RFP-cortactin WT (Fig. 9B). Importantly, transfection of RFP-cortactin 3YF exhibited transient co-localization with GFP-FAK WT at peripheral adhesions (Fig. 8B and Video S8), resulted in significantly increased FA lifetime (40 min, p<0.001) (Fig. 9A), and significantly decreased (<0.002 µm/sec, p<0.001) cell movement (Fig. 9B) compared to RFP-cortactin WT transfected cells. These results support the importance of cortactin tyrosine phosphorylation downstream of FAK in mediating enhanced FA dynamics.

Since FAK activity is important in promoting FA turnover [18] and GFP-FAK KD MEFs exhibit increased FA lifetime compared GFP-FAK WT MEFs (Fig. 2), GFP-FAK KD MEFs were transfected with RFP-cortactin WT or 3YE and were evaluated for effects on adhesion lifetime and cell motility (Figs. 8B and 9). Although RFP-cortactin WT transiently co-localized with GFP-FAK KD at peripheral adhesions (Fig. 8B and Video S9), this did not increase FA turnover or cell movement (Fig. 9). Moreover, expression of RFP-cortactin 3YE strongly localized to cell protrusions (Fig. 8B and Video S10), but did not promote FA turnover or cell motility of GFP-FAK KD MEFs (Fig. 9). Similarly, neither RFP-cortactin WT or 3YE over-expression in GFP-FAK A712/713 MEFs were able to increase FA turnover or cell speed (Figs. 8B and 9 and Videos S11 and S12). Although studies have shown that cortactin 3YE expression can enhance cell motility [38], this potential phospho-mimetic cortactin mutant does not rescue FA and motility defects of GFP-FAK KD or A712/713 MEFs. Together, with the effects of cortactin 3YF over-expression in preventing FA turnover and inhibiting cell movement, our results support the notion that FAK-mediated FA remodeling may occur through the formation of a FAK-cortactin signaling complex.

## Discussion

FAK is a signaling and scaffold-like protein regulating cell motility through diverse mechanisms [19]. Loss of FAK expression results in cells with FA turnover and motility defects. FAK has been proposed to regulate FA dynamics via recruitment of proteases [48], [49], via enhancement of integrin internalization [50], by interactions with talin [13], via regulation of Rho-family GTPase activity [51], and by phosphorylation of integrin-associated adaptor proteins [16]. Although the FAK N-terminal FERM domain has been shown to interact with actin regulatory proteins N-WASP [24] and Arp3 [52], we find that FAK−/− MEFs reconstituted with GFP-FAK constructs containing inactivating mutations within the C-terminal PRR domains exhibit severe defects in FA turnover and cell movement. Notably, FAK constructs containing PRR mutations localize to FAs, exhibit normal levels of Y397 FAK phosphorylation, and would be expected to possess intact N-terminal FERM domain function. Thus, proteins associating with FAK via binding to FAK C-terminal PRR regions play key roles in promoting FA turnover and cell motility.

Herein, we identify cortactin as directly binding to FAK PRR regions via its SH3 domain. Using FAK-null fibroblasts stably reconstituted with various GFP-tagged FAK constructs, we find that FAK activity and FAK C-terminal PRR2 and PRR3 are required for FA turnover and cell motility. Cortactin binds directly to FAK PRR2 and PRR3 sites via its SH3 domain and cortactin knockdown prevents FA turnover and the release of GFP-FAK from FAs. FAK-cortactin binding occurs in fibroblasts held in suspension and is negatively-regulated during the initial phase (30 min.) of cell spreading on fibronectin associated with cortactin tyrosine phosphorylation. FAK can directly phosphorylate cortactin at Y421 and Y466, and these sites are also phosphorylated by Src and Abl/Arg tyrosine kinases. It remains undetermined which tyrosine kinase is responsible for fibronectin-initiated cortactin phosphorylation, but genetic inhibition of FAK activity or reconstitution of FAK-null fibroblasts with a FAK PRR2 mutant unable to bind cortactin prevent fibronectin-stimulated cortactin tyrosine phosphorylation.

Importantly, we show that over-expression of cortactin mutated at Y421, Y466, and Y482 to phenylalanine (3YF) prevents FAK-enhanced FA turnover and cell motility. Notably, cortactin transiently co-localizes with FAK at FAs within MEFs after 60 min on fibronectin and this co-localization is enhanced in kinase-inhibited FAK R454 MEFs but does not occur in FAK-null fibroblasts reconstituted with FAK-PRR2 or -PRR3 mutants. Cortactin does not localize to FAs in FAK-null fibroblasts. We hypothesize that the FAK-mediated recruitment of cortactin to FAs results in cortactin tyrosine phosphorylation and the subsequent dissociation of the FAK-cortactin complex.

The molecular mechanism(s) promoting FAK-cortactin dissociation remain unclear. Previous studies have shown that cortactin mutated at Y421, Y466, and Y482 to glutamic acid (3YE) exhibits less binding to FAK than cortactin WT or cortactin 3YF [38]. Co-immunoprecipitation experiments suggest that FAK may not associate with tyrosine-phosphorylated cortactin [39]. Cortactin tyrosine phosphorylation occurs near the cortactin SH3 domain and mutation or phosphorylation of these sites may alter protein conformation. However, we find that cortactin 3YE over-expression exhibited enhanced leading edge distribution, did not alter FA dynamics, and did not rescue FA turnover defects under conditions of inhibited FAK activity. This result may be related to the fact that cortactin 3YE does not bind FAK or that cortactin 3YE mutations do not recapitulate SH2 domain target protein binding to tyrosine-phosphorylated cortactin.

**Figure 10:**
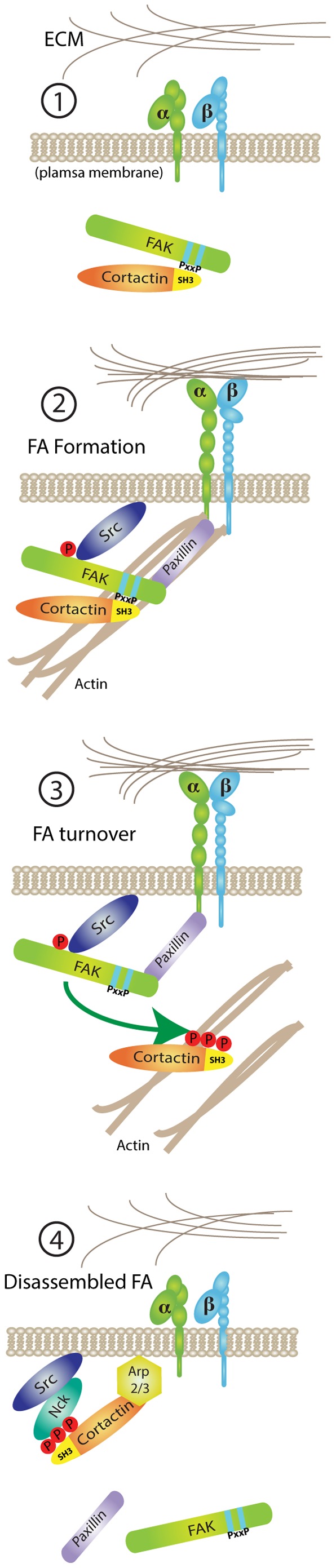
Model of FA turnover through FAK and cortactin association. 1) FAK and cortactin associate under anchorage-independent conditions. 2) Upon transmembrane integrin receptor clustering by cell replating on FN, cytoplasmic FAK is recruited and activated at newly forming focal adhesions (FAs). Cortactin SH3 domain binding to FAK C-terminal PRR2 and PRR3 facilitates transient cortactin localization to FAs resulting in alterations in FA-associated f-actin. 3) Direct or FAK-enhanced cortactin tyrosine phosphorylation results in FAK-cortactin complex dissociation associated with FA turnover and 4) the formation of other signaling complexes (such as Arp2/3 or the Nck adaptor protein) with tyrosine-phosphorylated cortactin.

Taken together, our results support a model (Fig. 10) whereby FAK-mediated FA remodeling may occur through the formation of a FAK-cortactin signaling complex. Our results are consistent with the importance of cortactin in promoting FA turnover in response to platelet-derived growth factor stimulation [8], with over-expression of cortactin 3YF in slowing FA turnover [9], and the importance of FAK C-terminal PRR domains in eliciting cortactin tyrosine phosphorylation associated with integrin-dependent *Staphylococcus aureus* uptake into cells [35].

So how can an actin regulatory protein like cortactin alter FA dynamics? Cell migration requires orchestrated changes in cell shape and FA interactions. Cell shape change is driven by altering f-actin cytoskeleton dynamics [4]. FA-associated proteins such as talin, vinculin and α-actinin bind f-actin and can link FAs to f-actin stress fibers. We speculate that FAK-mediated recruitment of cortactin to FAs may also act to regulate f-actin tethering at FAs. The importance of actin in stabilizing FAK localization to FAs is supported by findings that low concentrations of cytochalasin D or latrunculin A result in rapid loss of GFP-FAK from FAs and FA disassembly (A. Tomar, unpublished results). Thus, disengagement of f-actin from FAs is sufficient to cause FA turnover. Notably, previous studies showed that FAK phosphorylation of α-actinin reduces its f-actin binding activity [25]. Cortactin is as an f-actin cross-linking protein and tyrosine phosphorylation reduces cortactin f-actin binding and cross linking activity [53]. Additionally, cortactin tyrosine phosphorylation may alter binding interactions of the cortactin SH3 domain [54]. Thus simplistically, FAK-mediated recruitment of cortactin to FAs, followed by cortactin tyrosine phosphorylation, could facilitate f-actin disengagement, a reduction in localized tension at FAs, and a shift in equilibrium toward FA disassembly.

In summary, our results support a sequence of events involving FAK interactions with cortactin that promote FA turnover and cell migration. First, FAK is recruited to matrix-integrin sites via binding to integrin-associated proteins like paxillin. Cortactin binding to FAK C-terminal PRR2 and PRR3 facilitates the transient recruitment of cortactin to FAs resulting in the stabilization of adhesion-associated f-actin. Integrin-activated FAK promotes cortactin tyrosine phosphorylation that weakens binding between cortactin and FAK. Cortactin tyrosine phosphorylation could either occur directly by FAK or through the formation of a FAK-Src signaling complex [26] and results in destabilization of FAs that allows for changes in cell shape needed for efficient cell movement. Correspondingly, tyrosine-phosphorylated cortactin may be shuttled to membrane projections by binding to leading edge-associated proteins such as Src, Arg, or Nck [11], [55]. FAK PRR mutants are localized to FAs, but do not bind cortactin and act to prevent cortactin tyrosine phosphorylation. FAK PRR mutant-expressing cells exhibit slower FA turnover and decreased cell motility. Thus, FAK PRR-dependent recruitment and tyrosine phosphorylation of cortactin is likely associated with a cycle of FA stabilization, followed by FA turnover, and the initiation of leading edge membrane projections. The coordination of these molecular events facilitates directional cell movement.

## Materials and Methods

### Cells and Constructs

FAK−/− and FAK+/+ MEFs were maintained in growth media: Dulbecco’s Modified Eagle Medium (MEM) containing 10% FBS, 100 U/ml penicillin, 100 µg/ml streptomycin, MEM nonessential amino acids, and 1 mM sodium pyruvate) at 37°C in a 5% CO2 incubator as described [56]. Hemagglutinin (HA)-tagged FAK WT and FAK A712/713 in pCDNA3.1 were created as described [28]. pEGFP-C1 FAK A876/877 was created by ligating an EcoRI and SacI fragment from pCDNA3 FAK A876/877 [42] into pEGFP-C1 FAK and subcloned into pBabepuro as described [56]. All GFP-FAK constructs also contain a triple HA tag at the FAK C terminus. FAK−/− MEFs stably reconstituted with GFP-tagged FAK constructs (WT, R454, and A712/13) were created as described [56]. FAK−/− MEFs reconstituted with GFP-FAK A876/877 were infected with GFP-FAK retrovirus, selected in puromycin (2 µg/ml), and pooled populations of cells enriched by fluorescence-activated cell sorting as described [56]. Rat cortactin isoform B RFP-tagged cortactin WT, RFP-cortactin 3YF (Y384/429/445 to phenylalanine, F), and RFP-cortactin 3YE (Y384/429/445 to glutamic acid, E) were created as described [9]. Mouse cortactin GST-SH3 domain was created as described [11]. Full length GST-cortactin was generated by PCR, using 5′-aaaaaaggatccatgtggaaa-3′ and 5′-aaaaaactcgagctactgccg-3′ primers, and digested with BamHI and XhoI for subcloning into pGEX. GST-FAK fusion constructs for residues 853 to 946 of murine FAK were generated by PCR using primers 5′-aaaaaaggatccccagaagagtacgtc-3′ and 5′-aaaaaaatcgattcagtgtggccg-3′. GST-FAK 947–1052 was generated using 5′-aaaaaaggatccggtccgactggaaac-3′ and 5′-aaaaaaatcgattcaaggagctggatt-3′ primers. PCR products were digested with ClaI and BamHI for subcloning. FAK 686–1052 was created as described [42]. GFP-FAK fusion constructs with a C-terminus tandem affinity purification (TAP) tag 1–402, 396–686, and 411–686 were created as described [57]. All constructs were verified by DNA sequencing. mCherry paxillin was used as described [13]. Cells were transfected using JetPrime (Polyplus Transfection) per the manufacturer’s instructions.

### Antibodies, Reagents, and Transfection

Monoclonal antibodies (mAb)s to phosphotyrosine (clone 4G10), FAK (clone 4.47), and cortactin (clone 4F11) were from Millipore. Phospho-specific rabbit mab to FAK pY397 (44-625G) was from Invitrogen. HA tag (clone 16B12) and GFP (clone B34) mAbs were from Covance. p130Cas (clone 21) was from BD Biosciences. β-actin (clone AC-17) mAb, rabbit phospho-cortactin (pTyr421) polyclonal Ab, purified bovine plasma fibronectin, latrunculin A, cytochalasin D, and epidermal growth factor (EGF) were from Sigma-Aldrich. Mouse cortactin ON-TARGETplus SMART pool siRNA (L-044721-00-0005), mouse p130Cas ON-TARGETplus SMART pool siRNA (L-041961-00-0005), ON-TARGETplus Si Control (Scr) siRNA (D-001810-01), and siGLO transfection indicator were from Dharmacon-Thermo Scientific. The ON-TARGETplus non-targeting siRNA pools (Scrambled control) are designed, modified, and microarray-confirmed to have minimal targeting of known genes in human, mouse and rat cells according to the manufacturer. 100 pmol siRNA +100 pmol siGLO was used to transfect GFP-FAK WT reconstituted FAK−/− MEFs using Lipofectamine 2000 (Life Technologies). After 48 h, target knockdown was confirmed by immunoblotting.

### Cell Replating, Lysis, Immunoprecipitation, and Immunoblotting

For replating or imaging experiments, cells were starved (0.5% serum) 16 h at sub-confluent densities, treated with 0.06% trypsin and 2 mM EDTA in PBS (2.5 min at 37°C), trypsin was inactivated by addition of soybean trypsin inhibitor (0.5 mg/ml) with 0.25% bovine serum albumin (BSA) in DMEM, collected by centrifugation, resuspended in Migration Medium (DMEM with 0.5% BSA), and held at 37°C (2×10^5^ cells/ml) for 1 h. Acid-washed glass coverslips or plastic culture dishes were coated with FN (10 µg/ml in PBS) overnight, blocked with 1% BSA in PBS for 30 min, and pre-heated to 37°C prior to use in cell experiments. Total protein lysates were prepared at the indicated times in Extraction Buffer containing 1% Triton X-100, 0.5% sodium deoxycholate, and 0.1% SDS as described [56]. Cytochalasin-D (1 µM) and latrunculin-A (1 µM) were added to the lysis buffer to disrupt f-actin filaments and pre-cleared by agarose bead incubation. For immunoprecipitation, 2 µg of antibody was incubated with lysates (0.5 to 1.0 mg total protein) for 2 h at 4°C, collected by binding to protein G plus- or protein A-agarose beads, and washed three times in Extraction Buffer without SDS and sodium deoxycholate. SDS-PAGE, antibody immunoblotting, and sequential membrane reprobing was performed as previously described [58].

### Direct Protein Binding Assay

Prey proteins were in vitro translated using the TNT transcription–translation system (Promega). Expression constructs in pCDNA3 (1 µg) were translated in a mixture containing biotin-labeled lysine and diluted 50-fold into Triton Lysis Buffer (50 mM HEPES pH 7.4, 150 mM NaCl, 1% Triton X-100) for binding assays. Bait proteins were expressed as GST fusion proteins in bacteria (pGEX vector), pre-bound to glutathione-agarose beads, and 5 µg GST or GST fusion protein incubated (500 µL volume) with in vitro translated prey for 2 h at 4°C, washed three times in Triton Lysis Buffer, resolved by SDS-PAGE, transferred to polyvinylidene fluoride membranes whereby the bait protein was detected by Coomassie staining. The bound prey detected by streptavidin-horseradish peroxidase (HRP) immunoblotting.

### Chemotaxis Motility Assay

Cells were serum starved in 0.5% serum overnight at sub-confluent densities. Millicell chambers with 8 µm pores (Millipore) were coated on both sides of the membrane with 10 µg/ml FN in Migration Medium (0.5% BSA in DMEM) for 2 h. Membranes were washed with PBS and air dried for 2 h. Cells were suspended by limited trypsin-EDTA treatment. Soybean trypsin inhibitor (0.25 mg/ml in DME) was added, and cells were pelleted and washed in Migration Medium and enumerated (ViCell XR; Beckman Coulter). Cells were held in suspension for 1 h at 37°C, 5×104 cells in 0.3 ml were added to each Millicell chamber, units were placed into 24-well plates containing 0.4 ml growth media supplemented with 50 ng/ml EGF to stimulate chemotaxis, and cells were incubated for 4 h at 37°C. Cells were fixed, stained with crystal violet, and cells on the lower membrane surface were enumerated. Mean of values were obtained from three individual chambers from at least two independent experiments.

### Scratch-wound Motility Assay

12 well tissue culture plates were coated with FN (2 µg/ml) and cells were plated at a sub-confluent density (2.5×10^4^ cells per well) in growth media. After 24 h, cells were serum starved (0.5% FBS) overnight, wounded with a pipette tip, washed with PBS, and incubated in growth media supplemented with 50 ng/ml EGF and containing 0.5 µg/ml of mitomycin-C prior to imaging. For time-lapse wound healing experiments, images of cells in phase were acquired every 15 min in a humidified 5% CO2 environment at 37°C using an Olympus IX51 microscope, XY-controlled stage with Z focus drive (Olympus), 10X objective (UPLFL, 0.30 NA), a MAC5000 controller and LEP shutter (Ludl Electronics), and an OrcaER camera (Hamamatsu) controlled by Slidebook (v5.0) software. Wound closure percentage was calculated by the change in area between 0 and 10 h using Slidebook (v5.0) software. For each independent experiment, each experimental group was assayed in quadruplicates. At least 12 time-lapse image sequences were analyzed per group from 3 independent experiments.

### Immunofluorescent Staining

Cells were plated onto 10 µg/ml FN-coated glass coverslips in Migration Medium at 37°C for the indicated time, fixed in 3.7% paraformaldehyde for 15 min, and then permeabilized with 0.1% Triton X-100 for 10 min. Blocking was performed with 100 µg/ml ChromPure donkey IgG in PBS (Jackson ImmunoResearch Laboratories) at room temperature for 1 h. Cortactin (1∶100) or paxillin (1∶100) antibodies were diluted in PBS and incubated overnight at 4°C. After three washes in PBS, coverslips were incubated for 45 min in PBS containing rhodamine-conjugated donkey anti-mouse IgG (Jackson ImmunoResearch Laboratories) in cells expressing GFP fusion proteins, or FITC-conjugated donkey anti-mouse IgG (Jackson ImmunoResearch Laboratories) in cells expressing RFP-fusion proteins. Images were acquired at room temperature sequentially using a mercury lamp source, multiband dichroic, single-band exciter, and single band emitter filter sets (Chroma) on dual filter wheels, an Olympus IX81 spinning disc confocal at 60X (PlanApo, N.A. 1.42) and a OrcaER camera (Hamamatsu) controlled by Slidebook (v5.0) software. Image files shown were cropped, pseudo-colored, and contrast-adjusted using Adobe Photoshop CS3.

### Time-lapse Imaging and Quantification of Adhesion Lifetime

FAK−/−, FAK+/+, and GFP-FAK reconstituted FAK−/− MEFs either non-transfected, transfected with siRNAs, mCherry-paxillin, or RFP-cortactin constructs were plated at a sub-confluent density (50%) onto glass bottom dishes (MatTek) coated with FN (2 µg/ml), and serum starved overnight. Imaging of random migration was initiated after adding growth media supplemented with 50 ng/ml EGF and containing 0.5 µg/ml of mitomycin-C to prevent cell division. Images were acquired every 2 min for 1 h in a humidified 5% CO2 environment at 37°C using an Olympus IX81 with zero drift compensation focus control spinning disk confocal microscope at 60X (PlanApo, N.A. 1.42), using a mercury lamp source, multiband dichroic, single-band exciter, and single band emitter filter sets (Chroma) on dual filter wheels, and an OrcaER camera (Hamamatsu) controlled by Slidebook (v5.0) software. Quantification of adhesion lifetime was performed by background subtraction and pixel intensity analyses using Image J software (v1.38). Adhesion lifetime was determined by thresholding images to select for individual adhesions followed by tracking using an Image J (manual tracking) plug-in module. Kymographs obtained from GFP-FAK and RFP-cortactin co-expression were obtained by sequential GFP and RFP imaging and using the time-lapse tile view function in Slidebook (v5.0). Cell trajectories were obtained by tracking nuclear position over time enabling cell speed determination using Slidebook (v5.0). Image files shown were cropped, pseudo-colored, and contrast-adjusted using Adobe Photoshop CS3.

### Phosphorylation and Mass Spectrometry Analysis

Full-length recombinant GST-cortactin was phosphorylated in vitro in reactions containing recombinant FAK kinase domain as described [20]. In vitro phosphorylated GST-cortactin was in gel digested by trypsin followed by endoproteinase GluC using standard methods [59]. For LC-MS/MS analysis, peptides were analyzed using a QSTAR-Elite hybrid mass spectrometer (ABSCIEX) interfaced to a nanoscale reversed-phase high-pressure liquid chromatograph (Tempo) with a 10 cm-180 ID glass capillary packed containing 5-µm C18 ZorbaxTM beads (Agilent). Peptides were eluted from the C-18 column into the mass spectrometer using a linear gradient over 60 min at 400 µl/min. LC-MS/MS data were acquired in a data-dependent fashion by selecting the 6 most intense peaks with charge state of 2 to 4 that exceeds 20 counts, with exclusion of former target ions set to “360 seconds” and the mass tolerance for exclusion set to 100 ppm. Time-of-flight MS were acquired at m/z 400 to 1600 Da for 1 s with 12 time bins to sum. MS/MS data were acquired from m/z 50 to 2,000 Da by using “enhance all” and 24 time bins to sum, dynamic background subtract, automatic collision energy, and automatic MS/MS accumulation with the fragment intensity multiplier set to 6 and maximum accumulation set to 2 s before returning to the survey scan. Peptide identifications were made using paragon algorithm executed in Protein Pilot 2.0 (Life Technologies) and MASCOT® (Matrix Science).

### Statistical Analyses

A two-tailed unpaired Student’s t test was used to evaluate two groups. Significance between multiple groups was determined by one-way analysis of variance followed by either Dunnett’s or Tukey’s multiple comparison test.

## Supporting Information

Figure S1 **FAK phosphorylation of cortactin in vitro.** (A) Purified recombinant GST-cortactin was incubated in the presence (lanes 1, 2, 4 and 5) or absence (lane 3) of recombinant GST-FAK kinase domain. In vitro kinase reactions were evaluated in the presence (lanes 1, 3, 4 and 5) and absence (lane 2) of ATP and with (lane 4) or without 2-mercaptoethanol (lane 5). Reactions were separated by SDS-PAGE and visualized by anti-phospho-cortactin (pY421) immunoblotting or by Coomassie staining. (B) MS/MS spectra of the trypsin plus endoproteinase GluC-generated cortactin peptide AGSQQGLTYTSEPVYE phosphorylated at Tyr-466.
(TIF)

Video S1 **FA dynamics in GFP-FAK WT expressing cells.** Cells were plated at subconfluent density on FN coated (2 µg/ml) glass bottom dishes (MatTek), serum starved (12 h), treated with growth media supplemented with 50 ng/ml EGF and imaged every 2 min for 1 h. Images were acquired every 2 min for 1 h in a humidified 5% CO2 environment at 37°C using an Olympus IX81 spinning disk confocal microscope, XY-controlled stage with zero drift compensation focus control at 60X (PlanApo, N.A. 1.42), using a mercury lamp source, multiband dichroic, single-band exciter, and single band emitter filter sets (Chroma) on dual filter wheels, and an OrcaER camera (Hamamatsu) controlled by Slidebook (v5.0) software. Videos were created and compressed using QuickTime Pro (Apple). 30 sequential images are shown at 10 frames per second.
(MOV)

Video S2 **FA dynamics in GFP-FAK A712/713 expressing cells.** Cells were plated at subconfluent density on FN coated (2 µg/ml) glass bottom dishes (MatTek), serum starved (12 h), treated with growth media supplemented with 50 ng/ml EGF and imaged every 2 min for 1 h. Images were acquired as described for Video S1.
(MOV)

Video S3 **FA dynamics in GFP-FAK WT cells after Scr siRNA transfection.** GFP-FAK WT cells were transiently transfected with Scr siRNA (Dharmacon). 48 hour post-transfection cells were plated at subconfluent density on FN coated (2 µg/ml) glass bottom dishes (MatTek), serum starved (12 h), treated with growth media supplemented with 50 ng/ml EGF and imaged every 2 min for 1 h. Images were acquired as described for Video 1.
(MOV)

Video S4 **FA dynamics in GFP-FAK WT cells after siRNA mediated p130Cas knockdown.** GFP-FAK WT cells were transiently transfected with mouse p130Cas siRNA (Dharmacon). 48 h post-transfection, cells were plated at subconfluent density on FN coated (2 µg/ml) glass bottom dishes (MatTek), serum starved (12 h) and treated with growth media supplemented with 50 ng/ml EGF and imaged every 2 min for 1 h. Images were acquired as described for Video S1.
(MOV)

Video S5 **FA dynamics in GFP-FAK WT cells after siRNA mediated cortactin knockdown.** GFP-FAK WT cells were transiently transfected with mouse cortactin siRNA (Dharmacon). 48 hour post-transfection cells were plated at subconfluent density on FN coated (2 µg/ml) glass bottom dishes (MatTek), serum starved (12 h), treated with growth media supplemented with 50 ng/ml EGF and imaged every 2 min for 1 h. Images were acquired as described for Video S1.
(MOV)

Video S6 **FA dynamics in GFP-FAK WT cells transfected with RFP-cortactin WT.** 48 hour post-transfection cells were plated at subconfluent density on FN coated (2 µg/ml) glass bottom dishes (MatTek), serum starved (12 h), treated with growth media supplemented with 50 ng/ml EGF and imaged every 2 min for 1 h. Images were acquired as described for Video S1.
(MOV)

Video S7 **FA dynamics in GFP-FAK WT cells transfected with RFP-cortactin 3YE.** 48 hour post-transfection cells were plated at subconfluent density on FN coated (2 µg/ml) glass bottom dishes (MatTek), serum starved (12 h), treated with growth media supplemented with 50 ng/ml EGF and imaged every 2 min for 1 h. Images were acquired as described for Video S1.
(MOV)

Video S8 **FA dynamics in GFP-FAK WT cells transfected with RFP-cortactin 3YF.** 48 hour post-transfection cells were plated at subconfluent density on FN coated (2 µg/ml) glass bottom dishes (MatTek), serum starved (12 h), treated with growth media supplemented with 50 ng/ml EGF and imaged every 2 min for 1 h. Images were acquired as described for Video S1.
(MOV)

Video S9 **FA dynamics in GFP-FAK KD cells transfected with RFP-cortactin WT.** 48 hour post-transfection cells were plated at subconfluent density on FN coated (2 µg/ml) glass bottom dishes (MatTek), serum starved (12 h), treated with growth media supplemented with 50 ng/ml EGF and imaged every 2 min for 1 h. Images were acquired as described for Video S1.
(MOV)

Video S10 **FA dynamics in GFP-FAK KD cells transfected with RFP-cortactin 3YE.** 48 hour post-transfection cells were plated at subconfluent density on FN coated (2 µg/ml) glass bottom dishes (MatTek), serum starved (12 h), treated with growth media supplemented with 50 ng/ml EGF and imaged every 2 min for 1 h. Images were acquired as described for Video S1.
(MOV)

Video S11 **FA dynamics in GFP-FAK A712/713 cells transfected with RFP-cortactin WT.** 48 hour post-transfection cells were plated at subconfluent density on FN coated (2 µg/ml) glass bottom dishes (MatTek), serum starved (12 h), treated with growth media supplemented with 50 ng/ml EGF and imaged every 2 min for 1 h. Images were acquired as described for Video S1.
(MOV)

Video S12 **FA dynamics in GFP-FAK A712/713 cells transfected with RFP-cortactin 3YE.** 48 hour post-transfection cells were plated at subconfluent density on FN coated (2 µg/ml) glass bottom dishes (MatTek), serum starved (12 h), treated with growth media supplemented with 50 ng/ml EGF and imaged every 2 min for 1 h. Images were acquired as described for Video S1.
(MOV)
