# Supplementary figures and images for: Cortactin as a Target for FAK in the Regulation of Focal Adhesion Dynamics
Source: PLoS One. 2012 Aug 29;7(8):e44041. doi: 10.1371/journal.pone.0044041 (PMC3430618; doi:10.1371/journal.pone.0044041)

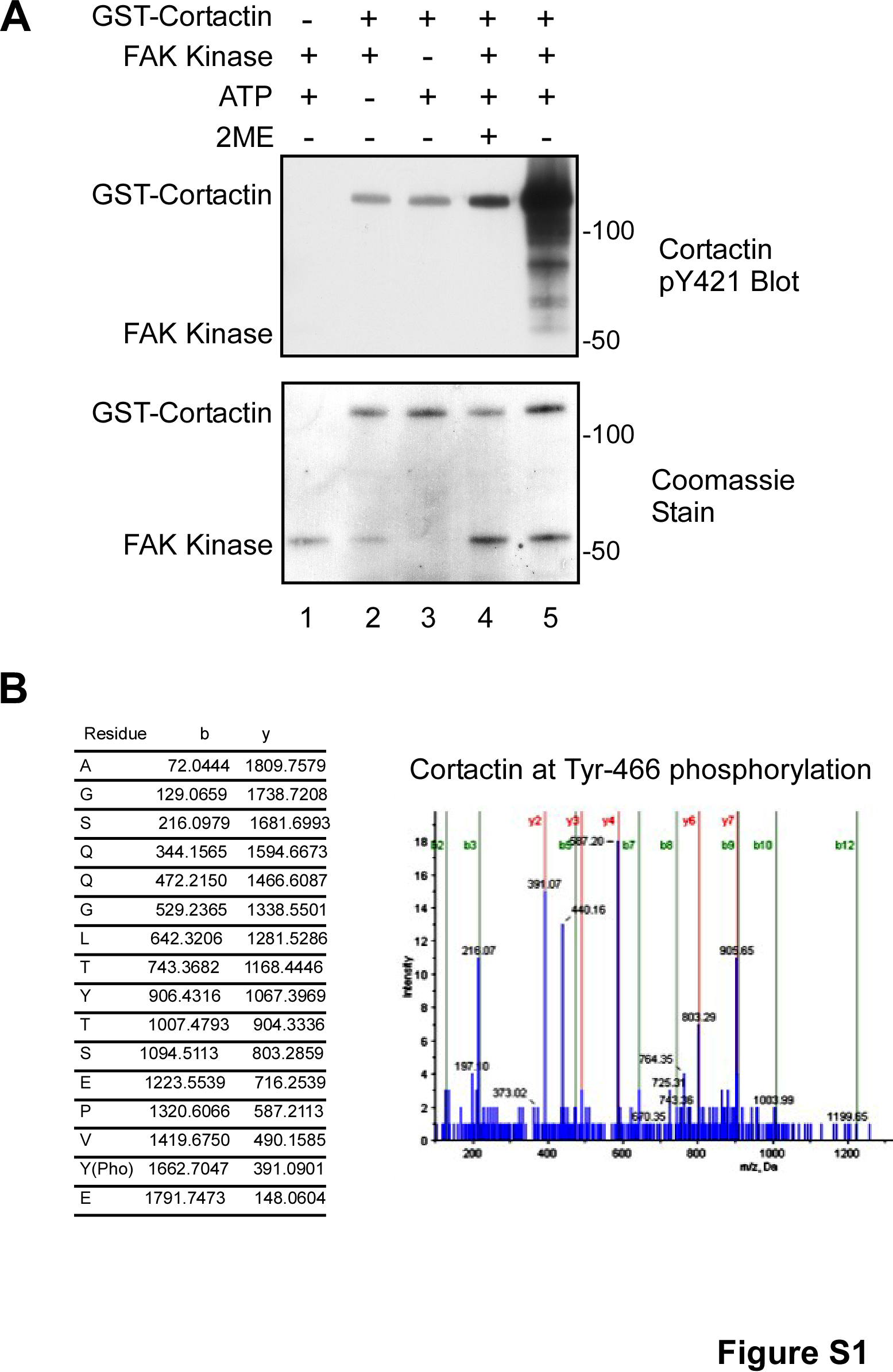

Supplement: Figure S1 — FAK phosphorylation of cortactin in vitro. (A) Purified recombinant GST-cortactin was incubated in the presence (lanes 1, 2, 4 and 5) or absence (lane 3) of recombinant GST-FAK kinase domain. In vitro kinase reactions were evaluated in the presence (lanes 1, 3, 4 and 5) and absence (lane 2) of ATP and with (lane 4) or without 2-mercaptoethanol (lane 5). Reactions were separated by SDS-PAGE and visualized by anti-phospho-cortactin (pY421) immunoblotting or by Coomassie staining. (B) MS/MS spectra of the trypsin plus endoproteinase GluC-generated cortactin peptide AGSQQGLTYTSEPVYE phosphorylated at Tyr-466. (TIF) [file pone.0044041.s001.tif]
